# CYPRI: a clinical decision-making tool to select psychiatric patients for pharmacogenetic testing

**DOI:** 10.1038/s41397-026-00414-4

**Published:** 2026-04-29

**Authors:** Ivana Tašková, Nicole Šafářová, Martina Hahn

**Affiliations:** 1 Department of Clinical Pharmacy, Psychiatric Hospital Bohnice, Prague, Czech Republic; 2 Department of Social and Clinical Pharmacy, Faculty of Pharmacy in Hradec Králové, Charles University, Prague, Czech Republic; 3 National Institute of Mental Health, Klecany, Czech Republic; 4 Third Faculty of Medicine, Charles University, Prague, Czech Republic; 5 Department of Psychiatry, Psychosomatics and Psychotherapy, University Medicine Frankfurt, Goethe University, Frankfurt am Main, Germany; 6 Department of Pharmacology and Clinical Pharmacy, Philipps-University Marburg, Marburg, Germany; 7 Department of Mental Health, varisano Hospital Frankfurt Hoechst, Frankfurt am Main, Germany

**Keywords:** Predictive markers, Psychiatric disorders

## Abstract

Pharmacogenetic (PGx) testing of cytochrome P450 (CYP) enzymes CYP2D6 and CYP2C19 is increasingly utilised to personalise psychopharmacological treatment, but clear criteria for patient selection are lacking. We developed and conducted a pilot evaluation of a clinical decision-making tool, the CYPRI (CYP Pharmacogenetic Risk Index), which uses routinely available clinical and pharmacological data to estimate the likelihood of a clinically relevant and actionable PGx testing result. A pilot observational study involving 34 patients was conducted at Psychiatric Hospital Bohnice in Prague, Czech Republic. A significant correlation was observed between the CYPRI score and the IMPACT score (ordinal logistic regression: t = 3.279, *p* = 0.0011; Kendall’s τ = 0.46, *p* = 0.0011). Discriminative ability was high, with an area under the curve (AUC) of 0.83 (95% CI: 0.69–0.97), based on ROC analysis performed using two outcome categories (0–2 vs. ≥ 2 points of IMPACT score). The optimal cut-off was 4. The CYPRI score offers a straightforward method to prioritise patients for PGx testing, potentially enhancing cost-effectiveness and clinical outcomes in psychiatric care.

## Background

Mental illnesses are among the top contributors to the worldwide disease burden and economic impact. The Global Burden of Disease study indicates that neuropsychiatric conditions consistently rank among the top ten causes of years lived with disability worldwide, with no significant improvement noted in recent decades, partially exacerbated by the COVID-19 pandemic [1–4].

Pharmacotherapy—alongside the psychotherapeutic approach—is a fundamental component of mental health treatment. However, its clinical effectiveness is often compromised due to an inadequate therapeutic response or the emergence of notable adverse drug reactions (ADRs). Reports indicate that treatment-resistant rates range from 20% to 60%, varying by diagnostic group and treatment context [5, 6].

Variability in drug responses and tolerability, often caused by differences in drug plasma levels, can partly be explained by genetic differences in metabolism, especially those involving cytochrome P450 (CYP) enzymes [7–9]. Among these, CYP2D6 and CYP2C19 are crucial in metabolising most psychotropic medications. Their high polymorphism rates make them key targets for pharmacogenetic (PGx) testing in psychiatry [7, 9–11]. The recent PRIME trial shows faster symptom improvement with PGx-guided treatment selection, but no significant differences in remission at 18 or 24 weeks [12]. Additionally, meta-analyses suggested that PGx-guided treatment may improve remission rates compared to treatment as usual [13, 14]. Consequently, genotyping of these enzymes is now widely used in clinical psychiatric practice. This marks a fundamental shift in psychiatric care, moving from a trial-and-error approach to more precise and personalised therapy. Several international organisations—such as the Dutch Pharmacogenetics Working Group (DPWG) [15], the Clinical Pharmacogenetics Implementation Consortium (CPIC) [16], the Canadian Pharmacogenomics Network for Drug Safety (CPNDS), and the French National Network (Réseau) of Pharmacogenetics (RNPGx)—have developed treatment guidelines to support the implementation of PGx testing into clinical practice [15, 17–20]. DPWG recommended preemptive testing for antidepressants in 2021 [15]. However, the other guidelines primarily focus on translating genetic findings into actionable prescribing decisions (gene-guided prescription) for relevant psychotropic drugs [9], without providing specific recommendations for identifying which patients are most likely to benefit from PGx testing. Consequently, psychiatric health care providers may remain unsure when to recommend PGx testing for their patients, which could lead to its overuse or underuse. There are no standard criteria for health insurance coverage of PGx testing. Different regulations apply across countries and sometimes even between insurance companies. The lack of a unified approach is one of the major barriers to implementing PGx effectively. A systematic search was conducted on PubMed using a combination of MeSH terms and free-text keywords to identify studies published since 2019 that proposed or evaluated clinical algorithms, decision-making tools, or criteria for selecting patients for PGx testing in psychiatry. Only 11 studies were retrieved, but none proposed a validated algorithm or clinical decision tool to support targeted testing. Although an expert statement by the International Society of Psychiatric Genetics (ISPG, 2019) and a narrative review by Stigl, Brockmöller, and Viviani (2013) suggest that PGx testing may be suitable following ADRs, toxicity, suicidality, treatment failure, or abnormal plasma levels, these recommendations remain vague and non-operational. To our knowledge, a structured approach has not yet been developed to systematically implement such guidance in a real-world clinical setting for selecting patients for PGx testing [21, 22].

Considering these limitations, the CYP Pharmacogenetic Risk Index (CYPRI) was developed as a practical and clinically relevant tool to identify psychiatric patients most likely to benefit from PGx testing, especially the need for PGx-guided medication adjustments. The aim of this study was to exploratorily evaluate the clinical effectiveness of CYPRI in detecting these patients and to propose a preliminary cut-off for the future validation studies.

## Methods

### Study and sample population

The pilot observational study was carried out at Psychiatric Hospital Bohnice in Prague, Czech Republic, from 31 January 2024 to 15 May 2025. It included 34 patients with various psychiatric diagnoses, comprising 29 inpatients and 5 outpatients referred through the clinical pharmacy consultation service (for more details, see Table S1).

### Inclusion criteria

Patients were referred for PGx testing by psychiatrists and/or clinical pharmacists, primarily for treatment-resistant conditions, unexpected therapeutic drug monitoring (TDM) results, or significant ADR occurrence. This retrospective pilot evaluation, therefore, included exclusively patients who had undergone PGx testing, all of whom would have had a CYPRI score of ≥1 if assessed prospectively.

### Description of CYPRI scoring system

The CYPRI scoring system was originally developed by clinical pharmacists at the Psychiatric Bohnice Hospital in Prague, Czech Republic, for internal purposes. It was created in the hospital environment, where high-quality PGx testing is available but costly, limiting its accessibility (for more details, see Table S2). CYPRI aims to identify patients who are likely to have altered CYP2D6 and CYP2C19 metabolism and to prioritise those for whom PGx testing could influence clinical decisions regarding medication (e.g., dose adjustment, medication change, or additional monitoring). The CYPRI criteria were developed by integrating clinical practice in psychiatric pharmacotherapy with evidence from the existing literature to indicate when PGx testing is most likely to be beneficial. Indicative features include treatment resistance, abnormalities in the TDM results (e.g., subtherapeutic or supratherapeutic/toxic drug plasma levels), and unexpected dose-concentration ratios (DCR) or parent drug metabolic ratios (MR) [7]. In addition, clinically relevant ADRs—particularly on initial or low doses of drugs metabolised by CYP2D6 and/or CYP2C19 and especially those that are concentration-dependent – were considered [8, 11, 21]. All selected criteria were used as indicators of the potential benefits of conducting PGx testing. Each criterion was assigned a point value of either 1 or 3, based on its estimated ability to identify patients with altered metabolic phenotypes related to CYP2D6 and/or CYP2C19 metabolism—specifically ultrarapid metabolizer (UM), rapid metabolizer (RM; in the case of CYP2C19 only), intermediate metabolizer (IM) and poor metabolizer (PM). Criteria supported by well-documented and robust evidence (e.g., TDM and ADRs) [8, 11, 21] were assigned 3 points, whereas those with less consistent or limited evidence were assigned 1 point. An overview of all criteria, including their score values and corresponding rationale, is presented in Table 1.

**Table 1:** CYPRI Score - Criteria and Point Allocation with corresponding rationale and reference.

POINTS	CRITERIA	DEFINITION
1	**The use of psychiatric medication that carries a high risk of variability in CYP2C19/CYP2D6 metabolism**	Psychiatric drugs are classified as CYP2D6/CYP2C19 substrates according to the Flockhart Table, Hiemke 2018 Consensus Guidelines for Therapeutic Drug Monitoring in Psychiatry, ClinPGx database, CPIC, and DPWG guidelines for PGx-based drug metabolism impact [7, 15, 16, 35, 36] Examples: *atomoxetine, modafinil, amitriptyline, nortriptyline, paroxetine, es-/citalopram, fluoxetine, aripiprazole, risperidone, diazepam, venlafaxine*.
1	**The use of somatic medication that carries a high risk of variability in CYP2C19/CYP2D6 metabolism**	Non-psychiatric drugs that are also substrates for CYP2D6 and CYP2C19 according to the Flockhart Table, ClinPGx database, CPIC, and DPWG guidelines [15, 16, 35, 36] Examples: *clopidogrel, codeine, tramadol, omeprazole, pantoprazole, ondansetron, metoclopramide, voriconazole, metoprolol, propafenone*.
1	**Treatment-resistant condition despite verified adherence and appropriate dosing**	Defined as the failure of two or more adequate medication trials [5].
3	**Drug plasma levels do not correlate with the administered dose**	Based on TDM results, defined as: ■ High dose with subtherapeutic drug levels (suggesting ultra-/rapid metabolism) or ■ Low dose with supratherapeutic/toxic drug levels (suggesting intermediate/poor metabolism) or ■ Plasma drug concentration falls outside the established minimum-maximum range (C/D_low_ and C/D_high_) for the specific drug according to Hiemke 2018 Consensus Guidelines for Therapeutic Drug Monitoring in Psychiatry or ■ Altered parent drug/metabolite ratio, indicating non-normal CYP2D6/CYP2C19 metabolism (e.g. *venlafaxine, risperidone, aripiprazole*) [7]. Clinically relevant interactions, phenoconversion, and severe hepatic or renal impairment must be excluded before scoring.
3	**Clinically significant adverse drug reactions (ADRs) CYP2D16/ CYP2C19 substrates as listed above**	Based solely on clinical observation and pharmacological anamnesis, the following criteria define an ADR: ■ Requiring dose adjustment, ■ Increased monitoring, ■ Drug discontinuation due to intolerance or toxicity, ■ Hospitalization due to ADR, ■ Information indication that the patient is unable to tolerate the drug despite standard dosing (reliable information in pharmacologic anamnesis e.g. medical opinion).
9	**TOTAL SCORE**	The total of all criteria determines patient eligibility for PGx testing.

### Description of outcomes (IMPACT Score)

Following the development of the CYPRI scoring system, it was necessary to conduct a pilot evaluation of its clinical utility by examining whether higher CYPRI scores were associated with more frequent medication adjustments, such as changes in drug selection or dosing, or the need for additional monitoring (e.g., ECG) due to an increased risk of ADRs. The IMPACT score was developed specifically for the purposes of this study, as no other method for assessing the clinical utility of CYPRI was available. Consequently, the IMPACT score has not yet been validated. It was designed to explore the potential clinical applicability of CYPRI, acknowledging that PGx test results alone do not necessarily warrant clinically relevant medication changes or additional monitoring.

Patients who gave informed consent underwent PGx testing. The clinical impact of the PGx testing results was then measured using the IMPACT score. Patients received 0, 1, or 2 points based on the clinical relevance of their PGx testing result. An additional 0.5 point was given for either a dual enzyme alteration or multiple medication adjustments (see Table 2 for details). The total IMPACT score was assessed to enable correlation with the overall CYPRI score. Given the limited sample size and to enhance the interpretability of regression trends, the original IMPACT scores were grouped into three clinically meaningful categories: no impact (total IMPACT score = 0), minor impact (1 or 1.5), and major impact (≥2). These categories were derived from the original IMPACT score (see Table 2).

**Table 2:** IMPACT Score – Criteria and Point Allocation, along with corresponding rationale.

POINTS	PRIMARY IMPACT CRITERIA	DEFINITION
**0**	**No impact**	The patient is a normal metabolizer for CYP2D6 and CYP2C19; no immediate intervention is required.
**1**	**Minor impact**	The patient is a non-normal metaboliser in CYP2D6 and/or CYP2C19; however, no intervention is required given the current medication regimen.
**2**	**Major impact**	The patient is a non-normal metaboliser in CYP2D6 and/or CYP2C19, requiring clinical action such as a change in medication, dose adjustment, or enhanced monitoring to prevent treatment failure or adverse effects.
**POINTS**	**MODIFYING FACTORS**	**DEFINITION**
**+ 0.5**	**Dual enzyme alteration**	The patient is a non-normal metaboliser in both CYP2D6 and CYP2C19, which could involve either decreased or increased enzyme function, resulting in a more complex pharmacogenetic impact.
**+ 0.5**	**Multiple medication adjustment**	An action was required in more than two medications.
**0–3**	**TOTAL SCORE**	The total of all criteria determines the effect of the PGx test result on the patient’s medication.

In most cases, these assigned impact categories corresponded closely with the need for clinical intervention, the primary criterion of the original IMPACT score. However, a shift from minor to major impact was only observed when patients accumulated points from both modifying factors (i.e., dual enzyme alteration and multiple medication adjustments), resulting in a score of 2. If only a single 0.5-point modifier was present, the patient remained within the minor impact category.

IMPACT summarised management actions observed after PGx results were available (dose adjustment, medication change, and additional monitoring). It was intended as a pragmatic proxy of clinical relevance; it does not imply that PGx alone triggered each action and was not used as a reference standard.

### Statistical analysis

The primary objective of the statistical analysis was to evaluate the predictive validity of the CYPRI score in identifying patients most likely to benefit from PGx testing, measured by the IMPACT score (i.e., medication adjustments or monitoring). Since the outcome variable (IMPACT score) is ordinal, ordinal logistic regression was employed with the total CYPRI score as a predictor. The proportional odds assumption was evaluated using the Brant test and was satisfied (*χ*2(1) = 2.91, *p* = 0.09), confirming the appropriateness of the model. The regression results were used to estimate the probability of PGx impact across three combined categories: no impact (IMPACT score = 0), minor impact (1 or 1.5), and major impact (≥2). To complement this analysis, a monotonic, non-parametric correlation using Kendall’s tau was performed. This method does not assume linearity and is robust for small sample sizes and tied values. While the ordinal regression examined the link between the CYPRI score and the categorised PGx impact levels (none, minor, major), Kendall’s tau assessed the relationship between individual CYPRI scores and the full range of the IMPACT score (0, 1, 1.5, 2, 2.5, and 3), preserving score granularity and enabling a complementary exploratory assessment of predictive consistency. All *p*-values were interpreted descriptively; values below 0.05 were considered strong evidence against the null hypothesis. A post-hoc power analysis was performed solely to address the sample adequacy. Cohen’s f² effect size was derived from the ordinal regression model using pseudo R². A simulation-based power analysis (cumulative logit; 2,000 replications, *n* = 34) was performed for the ordinal model to evaluate whether the available sample size provided sufficient power to detect the observed effect.

Finally, a Receiver Operating Characteristic (ROC) analysis was performed to assess the CYPRI score’s discriminative ability in distinguishing patients with major PGx impact (≥2 points) from those with no or minor impact (<2 points). The optimal cut-off point was identified using Youden’s index, which maximised the trade-off between sensitivity and specificity of the CYPRI scoring system. The final ROC curve was generated, and the corresponding area under the curve (AUC) was calculated. A generative CI-based power (binormal) analysis for the ROC (5000 simulations; binormal model; DeLong CIs) was also conducted.

All statistical analyses were performed using R software (version 4.4.2)

### Reporting guidance compliance

This observational study follows the Strengthening and Reporting Observational Studies in Epidemiology (STROBE) guidelines and the Academic Journals (AJ) Reporting Checklist for observational research.

## Results

The study sample comprised 34 participants, with a median age of 38 years (ranging from 17 to 69), and 76% were male. The most common primary diagnoses included schizophrenia spectrum disorders (41%, *n* = 14), anxiety disorders (21%, *n* = 7)—which covered generalised anxiety disorder and obsessive-compulsive disorder, bipolar and schizoaffective disorders (18%, *n* = 6), and intellectual disabilities alongside autistic spectrum disorders (9%, *n* = 3). Additionally, two patients with alcohol dependence participated (6%). Other diagnoses included major depressive disorder (3%, *n* = 1) and adjustment disorder (3%, *n* = 1) (for more details, see Table S1). To evaluate the predictive value of the CYPRI score in this population, we examined the relationship between the total CYPRI score (Table 1) and the clinical effects of PGx-guided medication adjustments—assessed by the IMPACT score (Table 2) —using two complementary statistical methods: ordinal logistic regression and Kendall’s tau rank correlation. Ordinal regression revealed a positive and statistically significant association (t = 3.279; SE = 0.267; *p* = 0.0011), indicating that patients with higher CYPRI scores were more likely to undergo substantial medication modifications (dose and drug changes) or additional monitoring, likely attributable to non-normal CYP2D6 and/or CYP2C19 metabolic phenotypes.

**Fig. 1:**
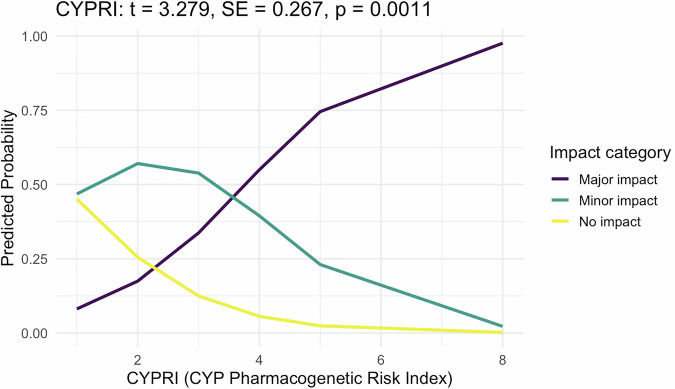
Predicted Probability of PGx Impact by CYPRI Score – Ordinal Regression Model (*n* = 34). In the regression model illustrated in Fig. 1, the total CYPRI score (Table 1) served as a predictor, and the clinical impact of the PGx test was categorised into three groups: no impact (IMPACT score 0), minor impact (IMPACT scores 1 and 1.5), and major impact (IMPACT score ≥2), according to the IMPACT scoring system outlined in Table 2.

**Fig. 2:**
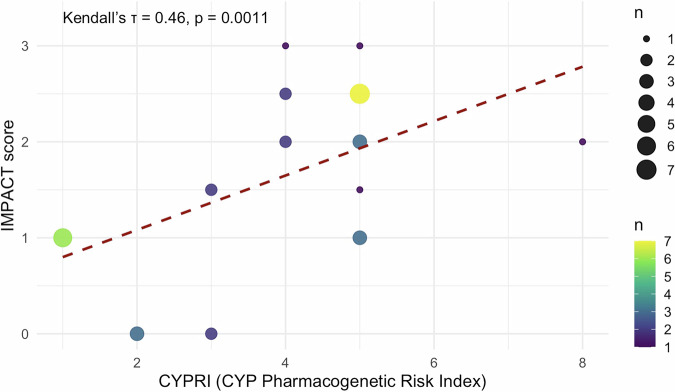
Bubble plot of CYPRI Score and IMPACT Score with Kendall’s Tau Regression Line (*n* = 34). Kendall’s tau correlation plot illustrates the relationship between the total CYPRI score and the total IMPACT score. Point size corresponds to the number of patients sharing the same value combination. Colour intensity indicates relative frequency using the viridis colour scale.

Alongside ordinal regression, a supplementary non-parametric analysis using Kendall’s tau correlation was performed. The ordinal regression model (see Fig. 1) evaluated the likelihood of categorical clinical impact based on increasing CYPRI scores. Conversely, Kendall’s tau correlation (see Fig. 2) assessed the monotonic relationship between the total CYPRI score and the full range of IMPACT scores (0 to 3), without categorisation. This approach maintained the ordinal nature of the data while enabling a more detailed examination of the consistency of the predictions. Likewise, Kendall’s tau analysis demonstrated a significant correlation between CYPRI and IMPACT scores (τ = 0.46, *p* = 0.0011), supporting the idea that higher CYPRI scores are associated with increased clinical relevance of PGx test results.

To assess the adequacy of the sample size in detecting the relationship between the CYPRI score and the effect of PGx testing, a post-hoc power analysis was conducted. Based on an ordinal regression model with a three-level categorisation of PGx impact (refer to the previous section), the effect size was calculated as Cohen’s *f²*=0.336, which corresponds to *R²* = 0.251. It was determined that at least 18 patients are necessary to achieve 80% power with a significance level of 0.05. Therefore, the current sample size of 34 patients meets this requirement, reinforcing the validity of the observed association (see Fig. 1). Consistently, a simulation-based power analysis for the ordinal regression model (cumulative logit; 2,000 replications) estimated 99.3% power (95% CI 0.989–0.997) at the current sample size of 34.

**Fig. 3:**
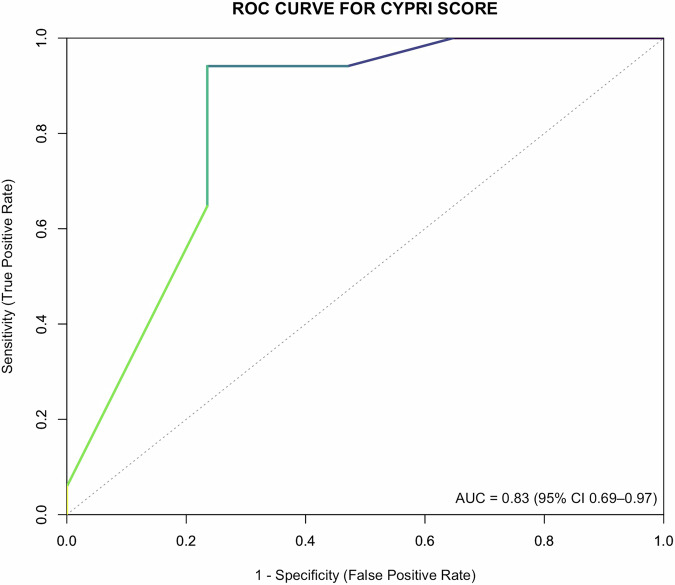
Receiver Operating Characteristic (ROC) curve for the CYPRI score. The area under the curve (AUC) was 0.83, with a 95% confidence interval of 0.69–0.97. The ROC analysis was based on binary classification (major versus non-major PGx impact; IMPACT score ≥2 versus 0–2) using a CYPRI cut-off 4 (Youden’s index optimum tied 3.5 and 4.0; integer cut-off reported as 4).

An ROC analysis was performed to evaluate the CYPRI score’s ability to differentiate between outcomes (binominal; 0–2 and ≥ 2 IMPACT scores) and to establish an optimal cut-off value. The AUC reached 0.83 (95% CI 0.69–0.97). However, the wide 95% confidence interval (0.69 to 0.97) reflects the limited sample size and highlights the exploratory nature of these findings (Fig. 3). We additionally performed a generative CI-based power analysis for the ROC (5000 simulations; binormal equal-variance model; DeLong 95% CIs) using our study sample (*n* = 34; balanced classes) and the observed AUC of 0.83 as the assumed true effect. The estimated power was 0.92 for demonstrating discrimination above chance (lower 95% CI > 0.50) and 0.31 for exceeding the minimum-effect threshold of AUC = 0.70 (lower 95% CI > 0.70). These results align with the wide empirical AUC CI and support an exploratory interpretation. The optimal CYPRI cut-off for identifying patients with significant PGx impact was determined using Youden’s index; because CYPRI is an integer score and the Youden optimum tied 3.5 and 4.0, we report cut-off 4. At this threshold sensitivity is 94% (95% CI 71.3–99.9%) and specificity is 76.5% (95% CI 50.1–93.2%). Therefore, patients scoring 4 or higher on the CYPRI are very likely to receive clinically meaningful PGx test results.

## Discussion

This study offers promising evidence for the effectiveness of the CYPRI scoring system as a practical tool to identify psychiatric patients most likely to benefit from PGx testing, particularly CYP2D6 and CYP2C19 genotyping in the context of the PGx-guided medication adjustments. Both ordinal logistic regression and Kendall’s tau rank correlation analyses revealed a consistent and statistically significant link between higher CYPRI scores and greater clinical impact of PGx test results. This was demonstrated by changes in medication, dose adjustments, or improved monitoring aimed at preventing treatment failure or ADRs, as evidenced by higher IMPACT scores. The ROC analysis further confirmed the strong predictive ability of the CYPRI score (AUC = 0.83; DeLong 95% CI 0.69–0.97) with an operating cut-off of 4. At this threshold, the score achieved 94% sensitivity (95% CI 71.3–99.9%) and 76.5% specificity (95% CI 50.1–93.2%), indicating good demonstrative performance in this pilot study. Although the ROC analysis was underpowered for exceeding the minimum-effect threshold of AUC = 0.70. Therefore, these findings indicate that the CYPRI score may help prioritise patients who are more likely to obtain clinically meaningful PGx testing results. Given the limited sample size, these conclusions should be interpreted cautiously and validated in larger cohorts.

Although interest in PGx testing—especially CYP2D6 and CYP2C19 genotyping—is growing in psychiatry, the overall evidence regarding its clinical and cost-effectiveness remains mixed. The strongest clinical benefits have been observed in the treatment of depression, where gene-guided therapy has enhanced treatment outcomes, including higher remission and response rates [23–27], as well as fewer adverse drug reactions, shorter durations of hospitalisation, and reduced treatment costs [28, 29]. In contrast, the clinical benefits of PGx-guided prescribing of antipsychotics have been less consistent [28, 30, 31]. Evidence is also more limited for other classes of psychotropic medications, aside from atomoxetine [15, 16]. Furthermore, assessing the cost-effectiveness of gene-guided therapy in psychiatry has been challenging due to small sample sizes, heterogeneity in study designs, methodological inconsistencies, and challenges in determining appropriate cost-effectiveness threshold [23, 32]. Notably, these mixed findings may partly result from most available studies being conducted in unselected populations, without using clinical criteria to categorise individuals by their likelihood of benefiting from PGx testing [24–27, 30–34]. In this context, the CYPRI offers a novel contribution aimed at bridging this implementation gap. It utilises routine clinical and pharmacological observations, such as previous ADRs, TDM results, or the status of treatment resistance, to estimate the pre-test probability that PGx testing will generate clinically actionable findings. This approach aims to support clinical judgement rather than replace it, offering a clear framework for prioritising patients for PGx testing in psychiatric care. The CYPRI is based on real-world clinical practice and has demonstrated a consistent statistical link between higher CYPRI scores and greater clinical impact of PGx test results. This emphasises its capacity to facilitate more targeted PGx testing implementation.

### Limitations

This study has several limitations. Most importantly, the exploratory, pilot nature of this study does not constitute a definite validation of the CYPRI scoring system. Moreover, this study was conducted in a single psychiatric hospital, which may limit the generalisability of the findings to other clinical settings or patient populations. Some CYPRI criteria—such as the evaluation of past ADRs or treatment resistance—partly rely on subjective clinical judgement and may be influenced by inter-rater variability. Another limitation is that both the predictor (CYPRI score) and outcome (IMPACT score) were assessed and developed by members of the study team, which could have introduced bias, despite the use of structured and predefined scoring criteria. Therefore, the IMPACT score was not intended or used as a reference standard, as it had not been validated. We also excluded patients who would have had a total CYPRI score of zero if assessed prospectively. Since this was a retrospective pilot study, only patients who had already undergone PGx testing could be included, and testing was performed only in those with a clear indication due to the limited availability and high costs of PGx testing. Therefore, patients who would have had a total CYPRI score of zero if assessed prospectively were excluded from this study. Because some CYPRI criteria, such as TDM-guided dose adjustments or ADR-driven medication switches, can occur independently of PGx testing, the observed relationship should be regarded as an association rather than a causal effect. Future validation should ideally be conducted across multiple centres, using independent raters and external patient cohorts, to confirm the robustness and generalisability of the findings.

Moreover, the CYPRI score is intended for settings with high-quality PGx testing; available assays often do not cover the full spectrum of CYP2D6 and CYP2C19 alleles. Therefore, the clinical impact predicted by CYPRI may not always be fully captured in these cases. Lastly, the study did not evaluate the usability and acceptability of the CYPRI scoring system among prescribing clinicians, which is essential for its implementation. However, informal clinical feedback suggests that a tool like this is needed in psychiatric practice.

## Conclusion

The CYPRI scoring system demonstrated, in this pilot study, its potential to serve as an effective clinical decision-making tool for identifying psychiatric patients who would most benefit from PGx testing, particularly CYP2D6 and CYP2C19 genotyping, in the context of the PGx-guided medication adjustments. By focusing on patients with significant treatment resistance, poor tolerability, or inadequate therapeutic response to psychotropic medication with high metabolic variability, the CYPRI score may support a rational and cost-effective approach to PGx testing. The findings demonstrated a statistically significant association between CYPRI scores and the clinical impact of PGx test results (measured as IMPACT score). Higher CYPRI scores—with a suggested cut-off of 4—were associated with a greater clinical impact, including larger medication adjustments and increased monitoring. These findings offer initial evidence for the predictive validity and potential clinical utility of the CYPRI score in psychiatric practice. In the future, the CYPRI score could function as a decision-making tool widely adopted by clinicians, patients, and healthcare insurers. Nevertheless, due to the exploratory and pilot nature of this study, additional validation in larger, prospective, and multi-centre cohorts is necessary before wider clinical adoption.

## Supplementary information

Table S1

Table S2

## Data Availability

Supplementary material is provided to improve transparency: Table S1 contains de-identified patient-level data, including demographics, diagnosis, medication, pharmacogenetic result, CYPRI scores, IMPACT scores, and phenotype-based categories. Table S2 provides details of the genotyping assay, including CYP2D6 and CYP2C19 variants (dbSNP ID, DNA and protein change, reference transcript, star-allele designations, and their tier classification).

## Data Origin and Visualisation Statement

The data analysed in this study originate from original clinical observations and internal analyses conducted within the study’s framework. All tables and figures included in the manuscript were created by the authors and have not been reproduced or modified from previously published works.
